# The role of TIGAR in nervous system diseases

**DOI:** 10.3389/fnagi.2022.1023161

**Published:** 2022-11-09

**Authors:** Bei Huang, Xiaoling Lang, Xihong Li

**Affiliations:** ^1^West China Second University Hospital, Sichuan University, Chengdu, China; ^2^Key Laboratory of Birth Defects and Related Diseases of Women and Children (Sichuan University), Ministry of Education, Chengdu, China

**Keywords:** TIGAR, stroke, PD, AD, seizures, brain tumors, nervous system diseases

## Abstract

TP53-induced glycolysis and apoptosis regulator (TIGAR) mainly regulates pentose phosphate pathway by inhibiting glycolysis, so as to synthesize ribose required by DNA, promote DNA damage repair and cell proliferation, maintain cell homeostasis and avoid body injury. Its physiological functions include anti-oxidative stress, reducing inflammation, maintaining mitochondrial function, inhibiting apoptosis, reducing autophagy etc. This paper reviews the research of TIGAR in neurological diseases, including stroke, Parkinson’s disease (PD), Alzheimer’s disease (AD), seizures and brain tumors, aiming to provide reference for the development of new therapeutic targets.

## Introduction

The TP53-induced glycolysis and apoptosis regulator (TIGAR), first reported in 2006 (Bensaad et al., 2006; Green and Chipuk, 2006), is a downstream target gene of p53 and an important factor involved in glycolysis and apoptosis (Hu et al., 2019b). It is indispensable in metabolism and is involved in metabolic syndrome, including hyperglycemia, insulin resistance, alcoholic fatty liver and tissue ischemia (Bensaad et al., 2006; Geng et al., 2018).

TIGAR is highly expressed in many cancer cells to promote cell survival (Bensaad et al., 2006, 2009; Xie et al., 2014; Yu et al., 2015). This may be related to its role in reducing reactive oxygen species (ROS), reducing DNA damage and improving DNA repair in these cells (Tang et al., 2021). TIGAR can aggravate myocardial ischemia injury and heart failure in animal and cell models (Kimata et al., 2010; Hoshino et al., 2012; Tang et al., 2021). In lung epithelial cells, PM2.5 exposure induces LKB1/p53/TIGAR dependent autophagy. TIGAR triggers autophagy, upregulates vascular endothelial growth factor (VEGF) transcription and protein synthesis, thereby aggravating chronic lung inflammation (Xu et al., 2019). TIGAR inhibits excessive autophagy and the degradation of tight junction proteins by promoting the flux of pentose phosphate pathway (PPP) and the production of nicotinamide adenine dinucleotide phosphate (NADPH), and then protects the structural integrity of tight junctions in cerebral microvascular endothelial cells (Wang et al., 2017a). Fructose-2,6-diphosphate (Fru-2,6-P_2_) can promote glycolysis and inhibit gluconeogenesis, normalizing the blood glucose level of diabetes animals (Monge et al., 1986; Rodríguez-Gil et al., 1991), while TIGAR can reduce Fru-2,6-P_2_ level and inhibit glycolysis, which may lead to elevated blood glucose and insulin resistance (Derdak et al., 2011). In 2015, the protective effect of TIGAR on cerebral ischemia–reperfusion injury and brain preconditioning was first reported (Zhou et al., 2016).

At present, the research on TIGAR in nervous system diseases is relatively rare, but targeting it may be a valuable strategy. TIGAR can increase the survival rate of stroke animals, improve motor function, and promote the recovery of cognitive function (Li et al., 2014). Because the oxidative damage of dopaminergic nigrostriatal neuronsis important in Parkinson’s disease (PD) pathogenesis, and TIGAR is significantly associated with neuronal glucose metabolism, PPP activity and oxidative stress, it may participate in the pathogenesis of PD (Tang et al., 2021). Decreased levels of TIGAR protein are found in different stages of Alzheimer’s disease (AD) dementia (Katsel et al., 2013). Oxidative stress and oxidative stress-induced neuronal apoptosis may be related to the occurrence and development of epilepsy, and TIGAR has antioxidant effect (Chen et al., 2019). TIGAR can also improve the survival rate of glioma cells under hypoxia (Wanka et al., 2012). This paper reviews researches on TIGAR in nervous system diseases, including stroke, PD, AD, seizures and brain tumors, and summarizes the potential role and mechanism of TIGAR, aiming to provide reference for the development of new therapeutic targets.

## *TIGAR* gene and protein

*TIGAR* gene is located on chromosome 12p13-3 and contains 6 exons and two p53 binding sites (Bensaad et al., 2006). One site is upstream of the first exon and the other is in the first intron (Bensaad et al., 2006). Transcription factors such as cyclic adenosine monophosphate (cAMP) response element binding protein (CREB) (Zou et al., 2013), SP1 (Sun et al., 2015), p53 (Wang et al., 2016), hypoxia-inducible factor-1α (HIF-1α) (Geng et al., 2018), hormones (Sun et al., 2015) and MicroRNA (Chen et al., 2015; Xu et al., 2017) can regulate the expression of TIGAR. (Table 1).

**Table 1:** Factors regulating the expression of TIGAR.

First author, year	Factors		Regulate the expression of TIGAR
Zou, S. 2013	Transcription factors	CREB	CREB regulates TIGAR expression through CRE binding sites on the TIGAR promoter. Knockout of *CREB* significantly decreased promoter activity and TIGAR expression in cells, while the overexpression of CREB enhanced promoter activity and TIGAR expression (Zou et al., 2013).
Sun, M. 2015		SP1	SP1 mediates TIGAR induction in ischemic neurons (Sun et al., 2015).
Wang, S.J. 2016		p53	The wild-type p53, p53^3KR^ and p53^4KR98^ can bind to the promoter of *TIGAR* gene (Wang et al., 2016).
Geng, J. 2018		HIF-1α	HIF-1α promotes TIGAR expression *via* binding to HRE in TIGAR promoter (Geng et al., 2018).
Sun, M. 2015	Hormones	Hormones regulating blood sugar levels or metabolism	Hydrocortisone, adrenaline and glucagon increased TIGAR expression. High insulin concentration promoted TIGAR protein expression, whereas low insulin concentration inhibited TIGAR protein expression (Sun et al., 2015).
Chen, S. 2015	MicroRNA	miR-144	The overexpression of miR-144 can inhibit TIGAR protein expression (Chen et al., 2015).
Xu, X. 2017		miR-101	Hypoxia induced upregulation of miR-101 expression, promoting glycolysis by targeting TIGAR mRNA (Xu et al., 2017).

CREB, cAMP-response element binding protein; CRE, cAMP-response element; HIF-1α, hypoxia-inducible factor-1α; HIF, hypoxia-inducible factor; HRE, HIF-responsive elements.

TIGAR protein is mainly found in the endoplasmic reticulum, cytoplasm, matrix and mitochondrial membrane of brain tissues (Geng et al., 2018). The comparison of the predicted amino acid sequences of TIGAR protein shows that it is highly conservative in vertebrates (Bensaad et al., 2006). TIGAR is functionally similar to the diphosphatase domain of 6-phosphofructo-2-kinase/fructose-2,6-bisphosphatase (PFK-2/FBPase-2) in regulating glycolysis, ROS level and cell apoptosis (Bensaad et al., 2006). TIGAR is a double phosphatase with two conserved pockets that bind phosphate molecules on the substrate by dephosphorylation (Geng et al., 2018). One pocket is located near the core histidine residues composed of His11, His183, Arg10, Arg61 and Glu89, and the other is located at the C-terminal near Arg10 and Asn217, according to the analysis of zebrafish TIGAR crystal structure (Geng et al., 2018). The two active sites of TIGAR are open, positively charged and can bind to other small molecules (Li and Jogl, 2009). TIGAR protein is abundant in brain (Li et al., 2014), heart (Okawa et al., 2019), kidney (Kim et al., 2015a), skeletal muscle (Geng et al., 2019) and a variety of cancer cells (Xie et al., 2014; Ma et al., 2017; Shen et al., 2018; Chu et al., 2020; Wang et al., 2020). Its expression level is high in olfactory bulb, cerebellum and cortex (Li et al., 2014).

## The physiological function of TIGAR

Previous studies have found that TIGAR can alleviate ischemic brain injury and play a neuroprotective role through reducing oxidative stress, inflammation, mitochondrial dysfunction, apoptosis and autophagy. (Table 2).

**Table 2:** The physiological function of TIGAR.

First author, year	Physiological function	Mechanisms
Li, M. 2014	Anti-oxidative stress	The PPP metabolic pathway regulated by *TIGAR* gene may be an effective mechanism against oxidative stress (Li et al., 2014).
Chen, J. 2018	Alleviating inflammation	TIGAR overexpression can inhibit the increase of inflammatory factors effectively, whereas *TIGAR* knockout has the opposite effect (Chen et al., 2018).
Li, Q. Q. 2021		NF-κB promotes inflammation by regulating the expression of a series of inflammation related genes, and TIGAR can inhibit NF-κB directly, preventing neuroinflammation (Li et al., 2021).
Giffard, R. G. 2005		TIGAR inhibits neuroinflammation *via* clearing ROS (Giffard and Swanson, 2005).
Feng, J. 2018	Maintaining mitochondrial function	TIGAR affected the integrity and degradation of mitochondria in 5-8F cells (Feng et al., 2018).
Geng, J. 2019		During the exhaustive exercise in mice, TIGAR was translocated to mitochondria and combined with ATP5A1 to protect mitochondrial function (Geng et al., 2019).
Li, M. 2014		The overexpression of TIGAR had a beneficial effect on mitochondria (Li et al., 2014).
Zhou, J. H. 2016	Inhibiting apoptosis	TIGAR reduces ROS-dependent apoptosis through PPP flux (Zhou et al., 2016).
Bensaad, K. 2009		ROS-dependent apoptosis was increased in TIGAR-deficient cells (Bensaad et al., 2009).
Bensaad, K. 2006	Reducing autophagy	TIGAR affects autophagy by regulating ROS, and the lack of TIGAR increases autophagy activity significantly (Bensaad et al., 2006).
Zhang, D. M. 2019		TIGAR may inhibit the activation of autophagy through the mTOR-S6KP70 signaling pathway (Zhang et al., 2019).
Bensaad, K. 2009		TIGAR is activated during the nutrient starvation of HeLa cells, inhibiting the induction of autophagy (Bensaad et al., 2009).

PPP, pentose phosphate pathway; NF-κB, Nuclear factor kappaB; ATP5A1, ATP synthase F1 subunit α; ROS, reactive oxygen species; mTOR, mammalian target of rapamycin.

### Anti-oxidative stress

Oxidative stress is characterized by excessive production of ROS and reactive nitrogen species (RNS) under harmful stimulation, leading to tissue damage and neuronal death (Kleinschnitz et al., 2010; Jiang et al., 2018). After ischemia/reperfusion (I/R), the inhibition of ROS has neuroprotective effect (Crack and Taylor, 2005). ROS is mainly generated from mitochondria (Niizuma et al., 2009) and NADPH oxidase (NOX) family-mediated reactions (Bedard and Krause, 2007)，applying oxidative stimulation to proteins, DNA, lipids and organelles in cells (Rahman, 2007; Kalogeris et al., 2012). NOX is the only known enzyme family acting exclusively for ROS production (Opitz et al., 2007). It transfers electrons from NADPH to molecular oxygen and then produces superoxide (Li et al., 2014). Four rodent genes catalyzing the subunit NOX have been identified, namely *Nox1*, *Nox2*, *Nox3* and *Nox4* (Kleinschnitz et al., 2010). Among them, NOX1, NOX2 and NOX4 are the main subtypes of NOX in the central system (Liu et al., 2020). NOX4-mediated oxidative stress led to neuronal damage through the leakage of blood–brain barrier and neuronal apoptosis (Kleinschnitz et al., 2010). The upregulation of NOX4 expression in striatum, especially mitochondria, increased the risk of oxidative stress (Liu et al., 2021). The induction of TIGAR was earlier than that of NOX4, and the application of NADPH could increase the content of glutathione (GSH) rather than ROS (Li et al., 2014). This indicated that the increase of endogenous NADPH caused by TIGAR upregulation would not produce more ROS through NOX4 under I/R conditions (Li et al., 2014).

The antioxidant activity of TIGAR is related to its regulation of glucose metabolism, maintenance of NADPH level, and regeneration of antioxidant GSH (Cheung et al., 2016). NADPH supplementation inhibited ROS and autophagy/lysosomal pathway (Liu et al., 2020). TIGAR can be activated by p53. It activates hexokinase and intracellular glucose metabolism shifts from glycolysis to PPP, leading to reduced ROS production and autophagy (Dodson et al., 2013). Under oxidative stress, neurons can use glucose to maintain their antioxidant state through PPP (Herrero-Mendez et al., 2009). PPP provides NADPH for antioxidant stress and energy supply (Li et al., 2014). Glucose-6-phosphate dehydrogenase (G6PD) is the rate limiting enzyme in PPP (Jain et al., 2004), and therefore, the level of G6PD would also determine the flux of PPP and the generation rate of NADPH (Li et al., 2014). After the overexpression of lentivirus-mediated *TIGAR* gene, G6PD was upregulated and PPP flux was increased, leading to a rise in NADPH production, and vice versa (Li et al., 2014). Overexpression of *TIGAR* gene can reduce intracellular ROS significantly. Thus, the PPP metabolic pathway regulated by *TIGAR* gene may be an effective mechanism against oxidative stress. Besides, neither G6PD silencing nor TIGAR mutation without enzymatic activity abolishes the antioxidative effects of TIGAR (Liu et al., 2022). After 6 h of OGD, both exogenous and mutant TIGAR failed to rescue NADPH loss in cells, reproducing the PPP deficiency observed in ischemic mouse brains, but both wild-type and mutant TIGAR significantly reversed ROS burst in cells (Liu et al., 2022). G6PD silencing did not eliminate the antioxidant and neuroprotective effects of TIGAR in cells with 6 h of OGD (Liu et al., 2022). These indicate that TIGAR has PPP-independent antioxidant activity in prolonged cerebral ischemia.

### Alleviating inflammation

Oxygen glucose deprivation/reoxygenation (OGD/R) or I/R induced the upregulation of inducible nitric oxide synthase (iNOS), cyclooxygenases 2 (COX2) and the releasing of proinflammatory cytokine interleukin 1 beta (IL-1β) and tumor necrosis factor-α (TNF-α) (Chen et al., 2018). TIGAR overexpression could inhibit the increase of these inflammatory factors effectively and *TIGAR* knockout had the opposite effect. This suggests that TIGAR may play an anti-inflammatory role by inhibiting the release of inflammatory factors and the activation of transcription factors.

Transcription factor nuclear factor kappaB (NF-κB) is the main regulator of ischemic injury severity. Reducing NF-κB activation can decrease inflammation after cerebral ischemia, and then alleviate brain injury (Dvoriantchikova et al., 2009). NF-κB promotes inflammation by regulating the expression of a series of inflammation-related genes, while TIGAR can inhibit NF-κB directly and inhibit NF-κB-mediated signals and inflammatory cytokines (Tang et al., 2018), preventing neuroinflammation (Li et al., 2021). NF-κB inhibitor protein (IκBα) is a protein inhibiting NF-κB. The level of total IκBα protein was decreased and NF-κB was increased significantly in cultured primary astrocytes during OGD/R prophase, accompanied by phosphorylation of IκBα and nuclear transmutation of NF-κB. TIGAR overexpression inhibited these changes, suggesting that TIGAR can alleviate inflammation by inhibiting the phosphorylation and degradation of IκBα and NF-κB expression (Chen et al., 2018). In addition, exogenous NADPH plays an anti-inflammatory role by inhibiting IκB kinase (IKK)/IκBα/NF-κB signal transduction (Qin et al., 2017), suggesting that TIGAR may inhibit NF-κB signal transduction by promoting NADPH production through PPP (Li et al., 2021).

The mechanism of TIGAR inhibiting neuroinflammation may be related to the clearance of ROS, because ROS is a powerful promoter of neuroinflammation (Giffard and Swanson, 2005). ROS stimulates the overexpression of proinflammatory genes such as TNF-α and IL-1β in the cerebral microvascular system, causing inflammatory responses (del Zoppo and Mabuchi, 2003; Tsai and Albers, 2015). ROS induces the activation of NOD-like receptor family pyrin domain containing 3 (NLRP3) inflammatory bodies (Minutoli et al., 2016). ROS can activate Toll-like receptor (TLRs) and peroxisome proliferator-activated receptors-γ (PPAR-γ), and microglia are sensitive to signals from TLRs and PPAR-γ (Kim et al., 2015b), triggering a series of inflammatory responses in turn. Studies have shown that TIGAR can increase the levels of NADPH and GSH, promoting the clearance of ROS (Quaegebeur et al., 2016).

### Maintaining mitochondrial function

As the motility of cells, mitochondria are critical in cellular energy homeostasis and also involved in regulating the different cell death mechanisms (He et al., 2020). Mitochondrial dysfunction is one of the markers of I/R damage, leading to neuronal death (Vosler et al., 2009). Fusion and division of membrane allow the mitochondrial contents to mix to maintain integrity, and the function of damaged mitochondria can be restored *via* fusion with adjacent intact mitochondria (Detmer and Chan, 2007).

TIGAR affected the integrity and degradation of mitochondria in 5-8F cells (Feng et al., 2018). Reduction of mitochondrial proteins and genes involved in mitochondrial biogenesis, replication, citric acid cycle, and fatty acid oxidation was observed in *TIGAR*-knockout mice (Geng et al., 2019). Mitochondrial TIGAR overexpression enhanced the generation of Adenosine triphosphate (ATP), maintained mitochondrial membrane potential and reduced mitochondrial oxidative stress under hypoxia condition (Geng et al., 2019). TIGAR increases exercise endurance, promotes remodeling of skeletal muscle, and regulates mitochondrial content and function through sirtuin 1 (SIRT1)-peroxisome proliferator-activated receptor γ coactivator 1α (PGC1α) pathways; in addition, during the exhaustive exercise in mice, TIGAR was translocated to mitochondria and combined with ATP synthase F1 subunit α (ATP5A1) to protect mitochondrial function (Geng et al., 2019).

TIGAR relocates to mitochondria under hypoxia, forms a complex with hexokinase 2 (HK2) on the outer membrane of mitochondria, and regulates the activity of HK2, helping to control the ROS of mitochondria (Cheung et al., 2012). Li et al. (Li et al., 2014) confirmed the localization of TIGAR in mitochondria and observed that the mitochondrial localization of TIGAR increased under the conditions of I/R and OGD/R, thereby believing that TIGAR could protect mitochondrial function. Their results showed that the knockout of *TIGAR* gene further reduced mitochondrial membrane potential, increased mitochondrial ROS and the activation of caspase-3, and triggered mitochondria-mediated apoptosis of damaged neurons (Li et al., 2014). Exogenous NADPH can reverse OGD-induced mitochondrial changes, caspase-3 activation and neuronal death resulting from *TIGAR* gene knockout. On the contrary, the overexpression of TIGAR had a beneficial effect on mitochondria (Li et al., 2014). Therefore, TIGAR protects neurons from I/R injury by clearing intracellular ROS and maintaining mitochondrial function.

### Inhibiting apoptosis

Apoptosis is a form of programmed death, which is crucial for the development and homeostasis of organisms (Birkinshaw and Czabotar, 2017). ROS is a key mediator of cellular oxidative stress and neuronal apoptosis during cerebral ischemia (Broughton et al., 2009). Its interaction with other tissue components produces a variety of other free radicals. Particularly, the interaction between O2-and NO produces highly toxic molecule peroxynitrite. This oxidant causes tissue damage and is one of the important trigger molecules of apoptosis (Wang et al., 2018). B cell lymphoma-2 (Bcl-2) family proteins are involved in the regulation of mitochondria-dependent apoptosis pathway (Gross et al., 1999). Anti-apoptotic protein Bcl-2 inhibits apoptosis *via* preventing mitochondrial membrane depolarization and inhibiting caspase-3 activation, while pro-apoptotic protein Bcl-2 associated protein X (Bax) promotes apoptosis *via* inducing mitochondrial membrane depolarization (Broughton et al., 2009). Bcl-2 participates in ischemic tolerance induced by preconditioning (Shimizu et al., 2001; Gwak et al., 2011). Isoflurane preconditioning (ISO) increases the expression of Bcl-2 to prevent the release of cytochrome c from mitochondria and inhibit neuronal apoptosis (Li et al., 2008). Bcl-2/B-cell lymphoma-extra large (Bcl-xL) may prevent the production of ROS in the process of apoptosis (Gottlieb et al., 2000). Bcl-2-deficient mice showed greater brain oxidative stress and sensitivity to oxidants than wild-type mice (Hochman et al., 1998). ROS may induce cell apoptosis through regulating Bcl-2/Bax-dependent mitochondrial apoptosis pathway (Zhou et al., 2016). ROS can also regulate Bcl-2 phosphorylation, ubiquitination, or cystine oxidation, resulting in decreased Bcl-2 expression and increased Bax expression (Li et al., 2004; Luanpitpong et al., 2013). The expression of TIGAR reduced ROS levels and thereby protected ROS-sensitive apoptotic responses (Bensaad et al., 2006). TIGAR cut down ROS-dependent apoptosis through PPP flux (Zhou et al., 2016). In TIGAR-deficient cells, ROS-dependent apoptosis were increased (Bensaad et al., 2009).

In addition, the activation of caspase is the most recognized biochemical feature in the early and late stage of apoptosis, and detection of active caspase-3 in cells and tissues is an important method to induce apoptosis by various apoptotic signals (Choudhary et al., 2015). Pretreatment of Zhou et al. (Zhou et al., 2016) inhibited the activation of caspase-3 in neurons induced by OGD/reperfusion, while *TIGAR* knockout increased the division of caspase-3, indicating that knockout of *TIGAR* gene can cancel the anti-apoptotic effect induced by pretreatment.

### Reducing autophagy

Autophagy is a cellular catabolic pathway that degrades and recycles proteins, damaged organelles, and misfolded proteins to maintain cell dynamic balance and normal cellular function (Wang et al., 2018). Moderate autophagy is neuroprotective, while excessive autophagy may lead to neuron cell damage in the central nervous system (CNS) (Shi et al., 2012). Autophagy impairment participates in the pathogenesis and progression of neurodegenerative diseases including PD (Zhu et al., 2022). Selective autophagy is an active mechanism in AD pathology, and regulating selective autophagy will be an effective strategy to control this pathogenesis (Guan et al., 2022). Proper PPP function and GSH levels are essential for normal autophagy (Dodson et al., 2013). The maintenance of GSH reduced form requires the participation of NADPH produced by PPP (Russell et al., 1999). TIGAR may raise GSH/oxidized glutathione (GSSG) ratio by increasing PPP flux while reducing glycolysis (Bensaad et al., 2006).

TIGAR play a neuroprotective role by inhibiting autophagy. Excessive ROS accumulation would disrupt the stable state of cells, not only causing oxidative stress, but also promoting the activation of autophagy (Bensaad et al., 2009). TIGAR affects autophagy by regulating ROS levels, and the lack of TIGAR can increase autophagy activity significantly (Bensaad et al., 2006). Severely damaged mitochondria are selectively removed by autophagy, called mitochondrial phagocytosis, to prevent apoptosis (Matsuda et al., 2010). BCL-2/adenovirus E1B 19KD interacting protein 3 (BNIP3) protein is an effective inducer of cell death and autophagy, causing a protective stress response to remove damaged mitochondria (Hamacher-Brady et al., 2007). Upregulation of TIGAR reduces BNIP3 activation by inhibiting ROS signal (Hoshino et al., 2012).

Mammalian target of rapamycin (mTOR) is the primary regulator of autophagy (Ghiglieri et al., 2010), and inhibiting mTOR-S6KP70 signaling pathway is one of the main signals to activate autophagy. Apart from reducing ROS, TIGAR also may limit autophagy activation through the mTOR-S6KP70 signaling pathway (Zhang et al., 2019). These suggest that autophagy inhibition is at least partially the basis of TIGAR’s neuroprotective mechanism in brain I/R-induced neuron injury (Zhang et al., 2019). How TIGAR regulates mTOR-S6KP70 signaling pathway remains to be further studied.

Beclin-1 protein is essential for autophagy (Xu and Qin, 2019). Microtubule-associated protein light chain 3 (LC3) is widely used to monitor autophagy, and the number of LC3-II is significantly related to that of autophagosomes (Mizushima and Yoshimori, 2007). After brain I/R in mice, autophagosomes were produced in large quantities, the expression of Beclin-1 and LC3II protein increased significantly, and p62 protein expression was significantly decreased, indicating that autophagy was activated after brain I/R (Zhang et al., 2019). After TIGAR overexpression, the expression levels of Beclin-1 and LC3II decreased significantly, and P62 expression increased, while the inhibition of *TIGAR* gene expression in mice had the opposite effect. Supplementation of autophagy inhibitor 3-methyladenine (3-MA) could reverse the damage caused by inhibiting *TIGAR* gene, indicating that the decrease of autophagy activation is related to the neuroprotective effect of TIGAR.

**Figure 1:**
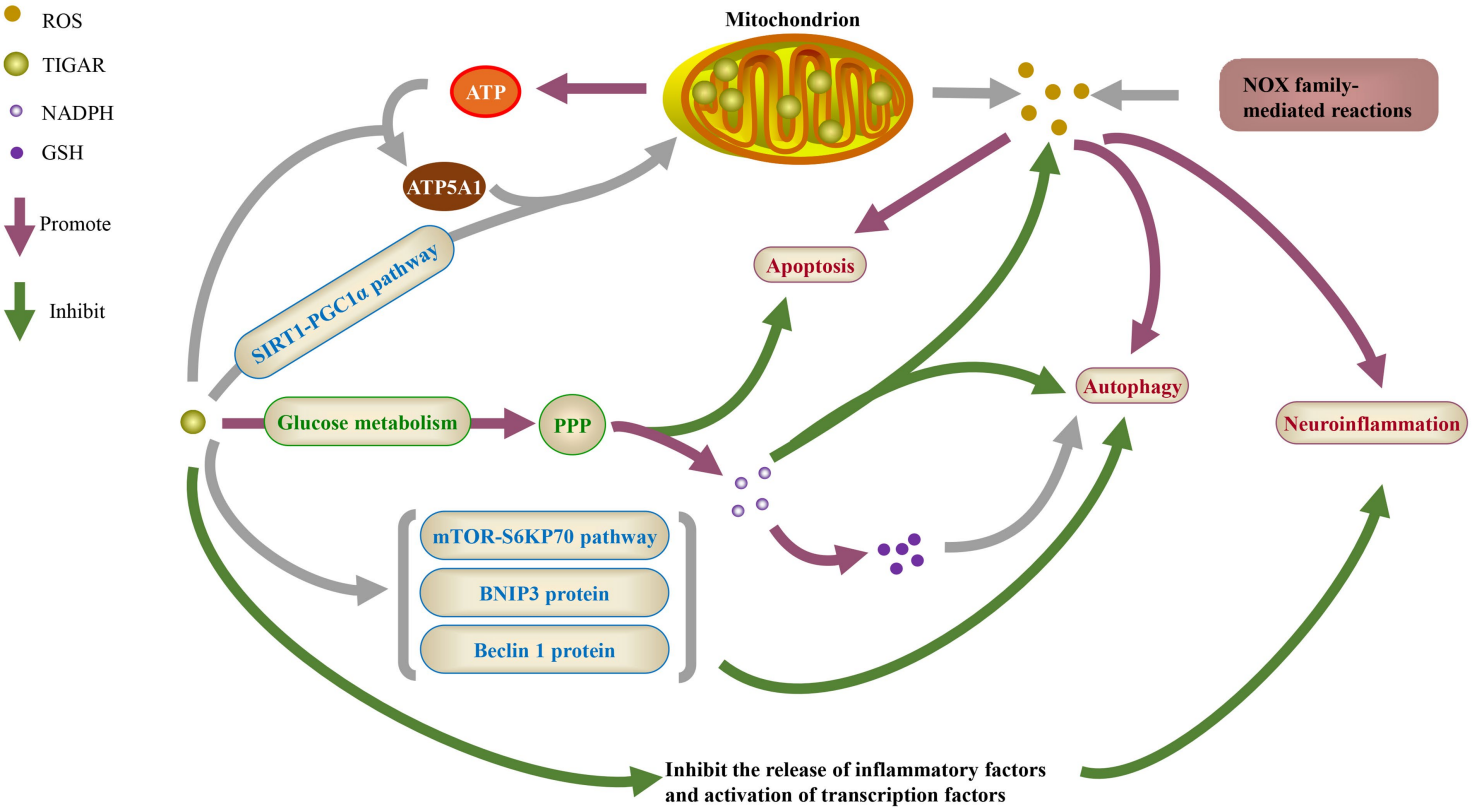
The physiological function of TIGAR. ROS, reactive oxygen species; NADPH, nicotinamide adenine dinucleotide phosphate; GSH, glutathione; ATP, adenosine triphosphate; ATP5A1, ATP synthase F1 subunit a; SIRT1, sirtuin 1; PGC1α, peroxisome proliferator-activated receptor γ coactivator 1α; PPP, pentose phosphate pathway; mTOR, mammalian target of rapamycin; BNIP3, BCL-2/adenovirus E1B 19KD interacting protein 3; NOX, NADPH oxidases.

In the lung tissue of patients with idiopathic pulmonary fibrosis, the level of TIGAR increased, which was associated with decreased LC3, p62, autophagosomes and overall autophagy flux (Patel et al., 2012). TIGAR was activated during nutrient starvation of HeLa cells, reducing reactive species and inhibiting the induction of autophagy (Bensaad et al., 2009). Thus, regulation of TIGAR has been proposed as a target for anticancer therapy, since blocking TIGAR activity can upregulate autophagy and improves the survival of cancer cells (Dewaele et al., 2010; Figure 1).

## The role of TIGAR In nervous system diseases

TIGAR plays an important role in nervous system diseases due to its multiple physiological functions. Its mechanisms in the occurrence and development of stroke, PD, AD, seizures and brain tumors provide references for treatment.

### Stroke

Stroke is caused by sudden interruption of cerebral blood flow (Chan, 1996), consisting of bleeding due to ruptured blood vessels and ischemia resulting from blocked vessels (Jiang et al., 2018). About 87% of strokes are ischemic (Benjamin et al., 2019).

Neuroinflammation is generally believed to cause brain damage or repair after strokes (Famakin, 2014). Astrocytes and microglia play crucial regulatory roles in the inflammatory response to ischemic injury. In ischemic conditions, astrocytes are activated and produce pro-inflammatory mediators and cytokines, including TNF-α, IL-1β and ROS (Giffard and Swanson, 2005; Iadecola and Anrather, 2011). In some cases, regulated astrocyte response may help repair nerve injury (Rossi et al., 2007). However, dysregulated astrocyte response or high levels of pro-inflammatory cytokines can be harmful to ischemic recovery by increasing the level of toxic nitric oxide and/or inducing neuronal apoptosis directly (Giffard and Swanson, 2005; Gelderblom et al., 2009). TIGAR can play an anti-inflammatory role by inhibiting the release of inflammatory factors and the activation of transcription factors (Chen et al., 2018).

NADPH, a metabolite of PPP, protects neurons from I/R-induced damage in rodent and primate stroke models (Li et al., 2016). GSH is the main source of sulfhydryl groups in most living cells and is of vital importance in maintaining the redox state of appropriate protein thiols, while NADPH can maintain the level of reduced GSH (Bensaad et al., 2006; Gu et al., 2015). ROS is a key mediator of cellular oxidative stress and neuronal apoptosis during cerebral ischemia (Broughton et al., 2009). I/R increased the production of ROS, applying oxidative stress to proteins, DNA, lipids and organelles in cells (Rahman, 2007; Kalogeris et al., 2012). TIGAR is relatively abundant in brain neurons and responds rapidly to I/R injury. The overexpression of TIGAR reduced OGD/R-induced astrocyte damage significantly, while *TIGAR* gene knockout aggravated such damage (Chen et al., 2018). The overexpression of adenovirus-mediated TIGAR not only reduced infarct volume, but also inhibited the decline of glial fibrillary acidic protein (GFAP) 24 h after reperfusion, reduced long-term mortality significantly and improved the recovery of neurological function *in vivo*, while *TIGAR* knockout had the opposite effect (Chen et al., 2018). TIGAR inhibit glycolysis and improve PPP as compensation, resulting in increased NADPH production, which helps to limit ROS (Bensaad et al., 2009; Cheung et al., 2012; Yin et al., 2014).

In animal and cellular stroke models, I/R increased mitochondrial localization of TIGAR (Li et al., 2014). TIGAR overexpression or NADPH supplementation saved OGD/R-induced ROS increase, GSH decrease, mitochondrial dysfunction and caspase-3 activation, which were exacerbated by *TIGAR* knockout (Li et al., 2014). The expression change and expression level of TIGAR during development may be related to the susceptibility of neurons to ischemic injury (Cao et al., 2015). Tripartite motif (TRIM) 31 is a new E3 ubiquitin ligase of TIGAR. TRIM31 deficiency alleviates mitochondrial damage induced by cerebral ischemia by upregulating TIGAR protein level (Zeng et al., 2021). TIGAR protects against ischemic brain injury by enhancing PPP flux and maintaining mitochondrial function, so it may be a valuable therapeutic target for ischemic brain injury (Li et al., 2014). Besides, TIGAR participates in ischemic preconditioning by clearing ROS and inhibiting neuronal apoptosis (Zhou et al., 2016). The combination of TIGAR and cerebral ischemic preconditioning also may be a new target for stroke prevention.

### PD

A key pathological feature of PD is the loss of dopaminergic (DA) neurons in substantia nigra (López et al., 2019). The lack of TIGAR protein in the pathological inclusion bodies of motor neurone disease (MND) or multiple system atrophy (MSA) indicates the specificity of disease, further enhancing the possibility that TIGAR may participate in the pathogenesis of PD (López et al., 2019). Apart from that, overexpression of mitochondrial HK2 conferred neuronal protection and survival in animal and cell models of PD and neurodegeneration (Gimenez-Cassina et al., 2009; Corona et al., 2010). Further studies are needed to elucidate TIGAR’s role in the pathogenesis of PD and figure out whether it is associated with hexokinase expression.

In the brain samples of PD patients, the progressive loss of DA neurons in the substantia nigra pars compacta (SNpc) is partly attributed to the defects in autophagy flux (Zatloukal et al., 2002; Tanji et al., 2011). Lack of DA neurons is associated with lysosomal dysfunction in PD (Wan et al., 2017). Reduced fusion between autophagosome and lysosome or reduced proteolysis in lysosomes leads to autophagy damage (Ge et al., 2021). Lysosome loss may cause PD, so restoring the function of lysosome seems to be a viable neuroprotective strategy for PD (Ge et al., 2021). Studies have shown that oxidative neurotoxin aroused lysosomal membrane permeabilization (LMP) in PD rodent model (Dehay et al., 2010; Wu et al., 2015), and selective autophagy is one of the protective processes concerning the clearance of damaged lysosomes (Papadopoulos et al., 2017). SP1-induced TIGAR could save the neurotoxin 1-methyl-4-phenyl-1, 2, 3, 6-tetrahydropyridine (MPTP)-induced lysosome dysfunction, avoiding the damage of DA neurons in PD (Ge et al., 2021). This identified that TIGAR-mediated lysosome repair may be a potential intervention for neurodegenerative diseases such as PD.

MPTP contributes to PD (Langston, 2017). Exogenous NADPH can prevent the toxicity of MPTP to dopaminergic neurons by increasing GSH, reducing ROS and inhibiting the neuroinflammation of substantia nigra compacta in mice (Zhou et al., 2018). Overexpression of G6PD in mouse dopaminergic neurons shows a protective effect against MPTP toxicity (Mejías et al., 2006). TIGAR may be a potential therapeutic target for PD by increasing the production of NADPH and G6PD.

### AD

AD is characterized by amyloid-β (Aβ) plaques and hyperphosphorylated Tau-associated neurofibrillary tangles in the brain (Durairajan et al., 2022; Iyaswamy et al., 2022). Oxidative stress, caused by the imbalance between ROS production and accumulation, is the main cause of Aβ-induced neurotoxicity in AD (Wang et al., 2022).

Studies have assessed TIGAR mRNA (*via* microarray) and protein levels in tissue homogenates of the superior temporal gyrus in patients with AD and control group after death (Katsel et al., 2013). Microarray data and protein levels assessed by westernblotting showed a decrease in TIGAR in AD patients compared with the control group.

Ataxia-telangiectasia mutated (ATM) can act as a functional component and effector of cell redox sensing (Cosentino et al., 2011). ATM and its downstream effector p53 are involved in tumor inhibition and the regulation of neural cell cycle (CCL), oxidative phosphorylation and glycolysis rate (Katsel et al., 2013). TIGAR protein levels decreased at different stages of AD dementia severity, indicating a diminished role of ATM-p53 signaling in countering glycolysis/oxygen-phosphorus-induced cell death (Katsel et al., 2013). TP53 activates TIGAR at low pressure (Madan et al., 2012). However, after long-term exposure to stress environment and induced TP53-mediated apoptosis, the expression of TIGAR decreased, suggesting that the induced apoptosis might reflect the loss of protection of survival signal induced by TP53 (Bensaad et al., 2006). Therefore, TIGAR may play a key role in the transformation of TP53-induced stress response, and its decreased expression may negatively affect cell survival during the progression of dementia (Katsel et al., 2013).

Hippocampal neurons are very important for the occurrence and development of AD (Hu et al., 2019a). AD is highly associated with neuroinflammation and oxidative stress in the brain leading to neuronal loss. The pro-inflammatory and redox-sensitive transcription factor NF-κB acts essentially in AD (Ju Hwang et al., 2019). MiR-146a-5pis deregulates in the peripheral blood of patients with AD and may serve as a biomarker for the diagnosis or treatment of AD (Gupta et al., 2017; Fransquet and Ryan, 2018). It is also involved in the progression from mild cognitive impairment to AD (Ansari et al., 2019). In AD patients, NF-κBis positively correlated with the expression level of miR-146a-5p, but there is a negative correlation between NF-κB mRNA and TIGAR mRNA, and between miR-146a-5p and TIGAR mRNA (Lei et al., 2021). NF-κB-induced upregulation of miR-146a-5p promoted oxidative stress and pyroptosis in hippocampal neuronal models of AD *via* TIGAR (Lei et al., 2021), which may be instructive to explore new therapeutic targets.

### Seizures

Oxidative stress in the brain can lead to neuronal apoptosis, leading to neurological diseases such as epilepsy thereby (Méndez-Armenta et al., 2014). Oxidative stress itself is the result of the first seizure and can trigger subsequent seizures (Patel, 2004; Puttachary et al., 2015). A growing body of evidence shows that overproduction of ROS in neurons disrupts intracellular Ca^2+^ homeostasis and leads to overload of calcium, causing neuronal hyperexcitation and the release of pro-apoptotic factors (Aguiar et al., 2012; Puttachary et al., 2015). The imbalance between ROS production and degradation would trigger oxidative stress, resulting in high ROS levels, which in turn damage macromolecules and cell membranes (Davies, 2000; Chrissobolis and Faraci, 2008).

In kainic acid (KA)-treated rats, TIGAR upregulation in cortex and hippocampus was accompanied by increased ROS levels, decreased antioxidant indices and increased pro-apoptotic factors. Increased TIGAR expression may be a feedback mechanism that promotes NADPH production and inhibits oxidative stress after seizures (Chen et al., 2019). TIGAR inhibits KA-induced seizures *via* inhibiting oxidative stress and neuronal apoptosis (Chen et al., 2019). Lentivirus-mediated TIGAR overexpression reduced KA-induced oxidative stress and neuronal apoptosis significantly, resulting in prolonged seizure latency, suggesting that TIGAR has anti-epileptic, antioxidant and anti-apoptotic effects, and therefore is a promising therapeutic target for seizures (Chen et al., 2019).

### Brain tumors

Glioblastoma multiforme (GBM), the most aggressive malignant brain tumor, is characterized by an imbalance between the growth of glioblastoma cells and glucose metabolism (Wang et al., 2017b). At present, it is highly resistant to treatment, including radiotherapy (Sinha et al., 2013). NFκB can be activated by ionizing radiation (IR) (Brach et al., 1991), while radiation-induced NFκB mediates radiation resistance in glioma cells by defending against oxidative stress (Iwanaga et al., 1998). The resistance to tumor necrosis factor-alpha (TNFα)-induced apoptosis of some tumors (Tsujimoto et al., 1985) is attributed to TNFα-mediated activation of NFκB (Beg and Baltimore, 1996; Wang et al., 1996). The radiation-induced cross effect of TNFα-NFκB promotes the survival of neuroblastoma cells (Veeraraghavan et al., 2011). NFκB enhances metabolic adaptation in cancer (Mauro et al., 2011), and P53 blocks activation of NFκB by inhibiting glycolysis (Kawauchi et al., 2008). P53 induction in these cells is regulated by TIGAR (Sinha et al., 2013). ROS sensitize glioma cells to chemotherapeutic agents (Sharma et al., 2007; Dixit et al., 2009), while TIGAR protects cells from ROS-related apoptosis (Sinha et al., 2013).

Gliomas, mainly composed of astrocytomas and oligodendrogliomas, are one of the most deadly cancers in the CNS (Giese et al., 2003). Haapasalo et al. (Haapasalo et al., 2003) analyzed 433 cases of astrocytoma and showed that the expression of thioredoxin reductase-1 (TrxR1) was upregulated in more than 66% of cases, which was significantly associated with relatively high proliferative activity and poor prognosis. Another study showed that immune reactivity of TrxR1 was observed in more than 50% of recurrent oligodendrogliomas, 1.5 times that of primary oligodendrogliomas, indicating that TrxR1 acts positively in the malignant progression of oligodendrogliomas (Järvelä et al., 2006). TrxR1 overexpresses in more than half of glioma patients (Haapasalo et al., 2003; Järvelä et al., 2006), so there is an urgent need for effective treatment of gliomas with TrxR1 overexpression. Overexpression of TrxR1 reduces the radiosensitivity of glioma cells significantly. Knockout of *TIGAR* gene can radio-sensitize TrxR1-overexpressed gliomas through inhibiting the nuclear transport of IR-induced thioredoxin-1 (Trx1) (Zhang et al., 2017). This suggests that elimination of TIGAR may be a strategy to radio-sensitize TrxR1-overexpressed gliomas.

TIGAR overexpressed in glioblastoma, and ectopic expression of TIGAR reduced cell death induced by glucose and oxygen restriction (Wanka et al., 2012). Metabolic analysis showed that TIGAR inhibited glycolysis and promoted respiration. Glioma cells benefit from TIGAR expression through raising energy production of glucose and enhancing defense mechanism against ROS *via* increasing respiration. This indicates that TIGAR is a major metabolic regulator, which can improve the survival rate of glioma cells under hypoxia (Wanka et al., 2012). Therefore, targeting metabolic regulators such as TIGAR may be a valuable strategy (Table 3).

**Table 3:** The role of TIGAR in nervous system diseases.

First author, year	Disease	Cause	The role of TIGAR	Species
Chen, 2018	Stroke	Neuroinflammation contributes to brain damage or repair after strokes (Famakin, 2014).	TIGAR plays an anti-inflammatory role by inhibiting the release of inflammatory factors and the activation of transcription factors (Chen et al., 2018).	Mice
Chen, 2018		ROS is a key mediator of cellular oxidative stress and neuronal apoptosis during cerebral ischemia (Broughton et al., 2009).	TIGAR-mediated overexpression reduced intracellular ROS in cultured primary astrocytes. (Chen et al., 2018).	Mice
Li, 2014		Cerebral ischemia causes stroke.	TIGAR protects against ischemic brain injury by enhancing PPP flux and maintaining mitochondrial function (Li et al., 2014).	Mice
Zeng, 2021		Cerebral ischemia causes stroke.	TRIM31 deficiency alleviated mitochondrial injury induced by cerebral ischemia by upregulating the level of TIGAR protein (Zeng et al., 2021).	Mice
Ge, 2021	PD	Lysosome loss may cause PD (Ge et al., 2021).	TIGAR could save MPTP-induced lysosome dysfunction (Ge et al., 2021).	Mice
Zhou, 2018, Mejías, 2006		MPTP contributes to PD (Langston, 2017).	NADPH prevents the toxicity of MPTP by increasing GSH, reducing ROS and inhibiting neuroinflammation (Zhou et al., 2018). Overexpression of G6PD shows a protective effect against MPTP toxicity (Mejías et al., 2006). TIGAR may be a potential therapeutic target for PD by increasing the production of NADPH and G6PD.	Mice
Katsel, 2013	AD	At different stages of AD dementia severity, the role of ATM-p53 signaling in countering glycolysis/oxygen-phosphorus-induced cell death diminished (Katsel et al., 2013).	TIGAR may play a key role in the transformation of TP53-induced stress response, and its decreased expression may negatively affect cell survival during the progression of dementia (Katsel et al., 2013).	Human
Lei, 2021		AD is highly associated with neuroinflammation and oxidative stress in the brain leading to neuronal loss.	NF-κB-induced upregulation of miR-146a-5p promoted oxidative stress and pyroptosis in hippocampal neuronal models of AD *via* TIGAR (Lei et al., 2021).	Human
Chen, 2019	Seizures	Oxidative stress in the brain can lead to neuronal apoptosis, leading to neurological diseases such as epilepsy thereby (Méndez-Armenta et al., 2014).	TIGAR inhibits KA-induced seizures *via* inhibiting oxidative stress and neuronal apoptosis (Chen et al., 2019).	Rats
Sinha, 2013	Brain tumors	The radiation-induced cross effect of TNFα-NFκB promotes the survival of neuroblastoma cells (Veeraraghavan et al., 2011).	NFκB enhances metabolic adaptation in cancer (Mauro et al., 2011), and P53 blocks activation of NFκB by inhibiting glycolysis (Kawauchi et al., 2008), P53 induction in these cells is regulated by TIGAR (Sinha et al., 2013).	Human
Zhang, 2017		Overexpression of TrxR1 reduces the radiosensitivity of glioma cells significantly.	Knockout of *TIGAR* gene can radio-sensitize TrxR1-overexpressed gliomas through inhibiting the nuclear transport of IR-induced Trx1 (Zhang et al., 2017).	Human
Wanka, 2012		Glucose and oxygen restriction induce cell death.	TIGAR can improve the survival rate of glioma cells under hypoxia (Wanka et al., 2012).	Human

ROS, reactive oxygen species; NADPH, nicotinamide adenine dinucleotide phosphate; PPP, pentose phosphate pathway; TRIM, tripartite motif; PD, Parkinson’s disease; MPTP, 1-methyl-4-phenyl-1, 2, 3, 6-tetrahydropyridine; GSH, glutathione; G6PD, glucose-6-phosphate dehydrogenase; AD, Alzheimer’s disease; ATM, Ataxia-telangiectasia mutated; NF-κB, Nuclear factor kappaB; KA, kainic acid; TNFα, tumor necrosis factor-alpha; TrxR1, thioredoxin reductase-1; IR, ionizing radiation; Trx1, thioredoxin-1.

## Conclusions and prospect

TIGAR plays an important role in antioxidant stress, reducing inflammation, maintaining mitochondrial function, inhibiting apoptosis, and reducing autophagy. Existing studies have shown that TIGAR may be a valuable therapeutic strategy in nervous system diseases. The combination of TIGAR and cerebral ischemic preconditioning probably become a new target for stroke prevention and treatment. TIGAR-mediated lysosomal repair also may be a potential intervention method for neurodegenerative diseases such as PD. The decrease of TIGAR expression may have a negative impact on cell survival during the progression of dementia. The increase of TIGAR expression is possibly a feedback mechanism to promote NADPH production and inhibit oxidative stress after epilepsy. TIGAR improve the survival rate of glioma cells under hypoxia.

At present, the research on TIGAR in nervous system diseases is relatively rare. There still remain many problems that need to be further clarified. The distribution of TIGAR in organelles, the regulatory mechanism of its expression, and the proteins interacting with TIGAR still need to be further studied. The regulatory mechanism of TIGAR repositioning in response to stress in the endoplasmic reticulum and nucleus is still unclear, which is worthy of further study. TIGAR’s ability to regulate ROS levels also plays a crucial role in autophagy, but the specific mechanism needs to be further explored. Overexpression of TIGAR inhibited autophagy activation and reduced ischemic brain damage in the one-hour/two-hour occlusion model. Further studies are needed to investigate the dynamic changes of autophagy and its role in ischemic stroke. Overexpression of HK2 can protect neurons in PD model. Further researches are expected to clarify the role of TIGAR in the pathogenesis of PD and figure out whether it is related to the expression of hexokinase. The molecular mechanism of oxidative stress and apoptosis in AD hippocampal neurons may have certain guiding significance for exploring new therapeutic targets, such as the overexpression of TIGAR, inhibition of oxidative stress and the knockdown of NF-κB or miR-146a-5p. The protective effect of TIGAR on seizures is not fully understood. Clinical application of lentivirus-mediated promotion of TIGAR overexpression still needs to be explored. Further studies are needed to elucidate the complex role and mechanism of TIGAR in the occurrence, development and treatment resistance of brain tumors. Although the treatment of nervous system diseases targeting TIGAR has great application potential, it is unknown that whether promoting its overexpression would affect other tissues and organs or not, because TIGAR is expressed in many tissues and organs throughout the body. The complex role of TIGAR in nervous system diseases and its neuroprotective effects through different mechanisms need to be further revealed, including potential agonists, inhibitors and related pathways, so as to provide references for the research of TIGAR-targeted drugs.

## Author contributions

BH drafted manuscript and prepared tables. XLi and XLa edited and revised manuscript. BH, XLa, and XLi approved final version of manuscript. All authors contributed to the article and approved the submitted version.

## Funding

This work was supported by The National Natural Science Foundation of China (82071353 to XLi); The National Key Research and Development Program of China (2017YFA 0104201 to XLi) and Key Research and Development Projects of Sichuan Province in China (2021YFS0029 to XLi).

## Conflict of interest

The authors declare that the research was conducted in the absence of any commercial or financial relationships that could be construed as a potential conflict of interest.

## Publisher’s note

All claims expressed in this article are solely those of the authors and do not necessarily represent those of their affiliated organizations, or those of the publisher, the editors and the reviewers. Any product that may be evaluated in this article, or claim that may be made by its manufacturer, is not guaranteed or endorsed by the publisher.
